# XmnI polymorphism is associated with fetal hemoglobin levels in hypoplastic syndromes

**DOI:** 10.1590/S1516-31802006000200012

**Published:** 2006-03-02

**Authors:** Marily Maria Azevedo Shimmoto, Perla Vicari, Andréa Cristina Fernandes, Gustavo Stuani Guimarães, Maria Stella Figueiredo

**Keywords:** Polymorphism (Genetics), Aplastic anemia, Paroxysmal hemoglobinuria, Fetal hemoglobin, Polymerase chain reaction, Polimorfismo (Genética), Anemia aplástica, Hemoglobinúria paroxística, Hemoglobina fetal, Reação em cadeia da polimerase

## Abstract

**CONTEXT AND OBJECTIVE:**

Acquired fetal hemoglobin (HbF) elevation has been implicated as a prognostic factor in dyserythropoietic disorders. Our objectives were to examine acquired HbF increases in aplastic anemia (AA) and paroxysmal nocturnal hemoglobinuria (PNH) patients, and to evaluate whether there is an association between the presence of XmnI and 5' hypersensitive site locus control region (LCR-HS2) polymorphisms and the HbF levels.

**DESIGN AND SETTING:**

Cross-sectional study at the Hematology and Blood Transfusion Service of Universidade Federal de São Paulo — Escola Paulista de Medicina.

**METHODS:**

We studied a group of 37 patients with AA and/or PNH. Polymerase chain reaction (PCR) and enzymatic digestion were utilized to analyze XmnI polymorphisms; and PCR, cloning and automated sequencing for the HS2 polymorphisms.

**RESULTS:**

The mean HbF level was 2.32%, but there was no significant difference in HbF level between the AA and PNH groups (p = 0.46). HbF levels of less than 1.0% showed a significant correlation with absence of the XmnI (+) polymorphism (p = 0.02). The presence of the XmnI allele was greater in the AA group (p = 0.007).

**CONCLUSIONS:**

XmnI polymorphism absence reduction is associated with acquired HbF elevation. Further studies are required to confirm these observations and make treatment, prognosis and survival comparisons.

## RESUMO

**CONTEXTO E OBJETIVO:**

O aumento adquirido da hemoglobina fetal (HbF) já foi implicado como fator prognóstico em distúrbios diseritropoiéticos. Nossos objetivos foram de examinar elevações adquiridas na HbF em pacientes com anemia aplástica (AA) e hemoglobinúria paroxística noturna (PNH), e de avaliar se há associação entre a presença de polimorfismos XmnI e de região de controle de locus gênico 5´ (LCR-HS2) e os níveis de HbF.

**TIPO DE ESTUDO E LOCAL:**

Estudo longitudinal no Serviço de Hematologia e Transfusão de Sangue da Universidade Federal de São Paulo – Escola Paulista de Medicina.

**MÉTODOS:**

Estudamos um grupo de 37 pacientes com AA e/ou PNH. Reação de polimerase em cadeia (PCR) e digestão enzimática foram usadas para analisar polimorfismos XmnI; e PCR para clonagem e sequenciamento automático dos polimorfismos HS2.

**RESULTADOS:**

O nível médio de HbF foi de 2,32%, mas não houve diferença significativa entre o nível de HbF dos pacientes AA e PNH (p = 0.46). Os níveis de HbF menores que 1,0% mostraram correlação estatisticamente significativa com ausência do polimorfismo XmnI (+) (p = 0.007).

**CONCLUSÕES:**

Ausência de polimorfismo XmnI está associado com diminuição de HbF. Mais estudos são necessários para confirmar estas observações e fazer comparações sobre tratamento, prognóstico e sobrevida.

## INTRODUCTION

There are several genetically different conditions under which higher levels of fetal hemoglobin (HbF) synthesis can persist into adult life. The clinical importance of the persistence of HbF expression in adults in relation to certain dyserythropoietic diseases remains controversial. However, acquired HbF elevation has been implicated as a prognostic factor in myelodysplastic syndrome (MDS) and aplastic anemia (AA).^1,2^

There are many factors that could change the level of HbF in adults, such as age, sex, polymorphisms, β-globin locus control region (LCR) and HbF persistence. Among the polymorphic genetic markers of the β-globin gene cluster, the XmnI polymorphism, a common C→T variation at position –158 of the ^G^γ globin gene, and a set of sequence variations in the 5' hypersensitive site (HS2) of the LCR have been associated with increased HbF levels in normal individuals, and in β-thalassemia and sickle cell anemia patients.^2,3^

## OBJECTIVE

Our objectives were to examine acquired HbF increases in AA and paroxysmal nocturnal hemoglobinuria (PNH) patients, and to evaluate whether there is an association between the presence of XmnI and 5' hypersensitive site locus control region (LCR-HS2) polymorphisms and the HbF levels.

## MATERIAL AND METHODS

### Setting

This was a cross-sectional study carried out within the Hematology and Blood Transfusion Service of Escola Paulista de Medicina, Universidade Federal de São Paulo, São Paulo, Brazil.

### Study plan

Thirty-seven patients were included in this study: 27 AA patients (13 females and 14 males) with a mean age of 39.44 years (range from 15 to 73); and 10 PNH patients (5 females and 5 males) with a mean age of 48.10 years (range from 22 to 81).

### Hematological and hemoglobin analysis

Hematological indices were determined using an automated cell counter. HbF was measured by alkali denaturation. The diagnoses of AA and PNH were based on clinical and laboratory features, including bone marrow biopsy, Ham's test and flow cytometry.

### DNA studies

The presence of XmnI polymorphism of the ^G^γ gene was established by means of the polymerase chain reaction, followed by digestion with XmnI. The nucleotide sequence of the LCR-HS2 region was determined by an allele-specific sequencing procedure.^3^

### Statistical analysis

All the results were investigated by statistical comparison using the Fisher test.

### Ethics

This study was approved by the Ethical Committee of Universidade Federal de São Paulo — Escola Paulista de Medicina (Unifesp-EPM).

## RESULTS

The percentages of patients with elevated HbF were 59% in the AA group and 40% in the PNH group. The mean HbF level in our study was 2.32% (standard deviation, SD: 2.47). There was no significant difference in HbF levels between the AA and PNH groups, which were 2.58 and 1.62% respectively (p = 0.46).

The time when the disease was diagnosed was unrelated to the HbF level. Similarly, no correlation was observed between length of time with the disease and XmnI polymorphism.

Analysis of DNA from both groups showed that there were twelve (44.45%) XmnI heterozygotes and three (11.12%) XmnI homozygotes in the AA group; and one (10.00%) heterozygote and one (10.00%) homozygote in the PNH group. The presence of the XmnI allele was significantly higher in the AA group (p = 0.007) (Table 1). The HbF level was compared with presence or absence of the XmnI allele, and it was found that HbF levels of less than 1.0% had a significant correlation with absence of the polymorphism (p = 0.02).

**Table 1:** XmnI allele polymorphism (-158 ^G^γ allele C/T polymorphism) analysis according to aplastic anemia group (AA) and paroxysmal nocturnal hemoglobinuria group (PNH); p = 0.007

	XmnI (+)		XmnI (-)		
Disease	n	(%)	n	(%)	Total
PNH	3	(4%)	17	(23%)	20 (27%)
AA	28	(38%)	26	(35%)	54 (73%)
**Total**	**31**	**(42%)**	**43**	**(58%)**	**74 (100%)**

*C = cytosine; T = thymine.*

The presence of LCR-HS2 polymorphism was observed in only one patient with AA. The patient was an African-Brazilian man with AA, presenting XmnI (-) and HbF of 6.2%. The polymorphism observed was the same as the Benin haplotype described in sickle cell disease, which could be associated with the patient's ethnic group.

## DISCUSSION

Elevated expression of HbF substantially benefits patients with β-globin disorders.^1^ On the other hand, increased HbF levels in acquired hemopathy has been associated with a better prognosis.^1,2^

In our study, the HbF level was considerably higher than expected, as was the frequency of patients with elevated HbF. The presence of hereditary persistence of fetal hemoglobin in Brazil has been shown to be approximately 0.01%, while we observed 59% and 40% in the AA and PNH patients, respectively.^4^ These data confirm the association between elevated HbF level and hematopoietic stress that was described previously.^2^

Although there is a need for better understanding of the meaning of the presence of HbF in adults, few reports have evaluated the importance of XmnI and LCR-HS2 polymorphisms in populations without hemoglobin disorders.^1-3^ We showed a XmnI polymorphism rate of 45.94% in our sample, with significant correlation with the HbF level. These results are consistent with previous Brazilian work on AA that showed higher ^G^γ values with higher HbF levels in bone marrow failure syndromes.^5^ However, no correlation with the length of time since the diagnosis was found.

## CONCLUSIONS

We conclude that XmnI polymorphism is probably implicated in acquired HbF elevation in hypoplastic syndromes. Further studies are required to confirm these observations and make treatment, prognosis and survival comparisons with regard to these hematological disorders.
